# Roles of Rac1-Dependent Intrinsic Forgetting in Memory-Related Brain Disorders: Demon or Angel

**DOI:** 10.3390/ijms241310736

**Published:** 2023-06-27

**Authors:** Wei Wang, Zixu Wang, Jing Cao, Yulan Dong, Yaoxing Chen

**Affiliations:** 1 Neurobiology Laboratory, College of Veterinary Medicine, China Agricultural University, Beijing 100193, China; 2 Key Laboratory of Precision Nutrition and Food Quality, Key Laboratory of Functional Dairy, Ministry of Education, Beijing Laboratory of Food Quality and Safety, Department of Nutrition and Health, China Agricultural University, Beijing 100083, China

**Keywords:** Rac1, intrinsic forgetting, Alzheimer’s disease, autistic symptoms

## Abstract

Animals are required to handle daily massive amounts of information in an ever-changing environment, and the resulting memories and experiences determine their survival and development, which is critical for adaptive evolution. However, intrinsic forgetting, which actively deletes irrelevant information, is equally important for memory acquisition and consolidation. Recently, it has been shown that Rac1 activity plays a key role in intrinsic forgetting, maintaining the balance of the brain’s memory management system in a controlled manner. In addition, dysfunctions of Rac1-dependent intrinsic forgetting may contribute to memory deficits in neurological and neurodegenerative diseases. Here, these new findings will provide insights into the neurobiology of memory and forgetting, pathological mechanisms and potential therapies for brain disorders that alter intrinsic forgetting mechanisms.

## 1. Introduction

Animal brains, including human beings, have the extraordinary capacity to constantly receive, store, integrate, and recall new information from the external world throughout their lifetime. Part of what animals learn is used for achieving self-state-driven or circumstance-driven behavioral flexibility. Without it, animals are condemned to an everlasting present. For instance, when animals learn that a stimulus, such as a poisonous food or a predator-infested environment, can generate aversion or danger, then their brains are capable of acquiring and storing this information in a way that encodes memory. Such a memory avoids repeating an unfriendly experience again in the future. From the perspective of neuroscience, a learning experience elicits the enduring offline chemical and/or physical modifications in the brain, which are also known as engrams [1]. These representations may be any changes in the physiological state and/or the excitability of certain neurons (engram cells) that are activated by a given learning, including adaptations in cellular signaling, ion channels, and synapses. For example, adaptations in cellular signaling can decrease or increase the neuron’s overall capability to receive, integrate, and process inputs from different types of cues. Changes in ion channels alter the inhibitory or excitatory properties of neurons, allowing them to conduct action potentials or other current signals. Changes in synapses mobilize neurite retraction or neuronal growth to dimmish or enhance the synaptic connectivity and strength between these neurons [2,3,4]. The collection of engram cell ensembles localized within different brain regions supports a memory that can be retrieved upon the subsequent presentation of appropriate cues present at the learning experience. As Susumu Tonegawa pointed out in his update of Hebb’s law, engram cells that fire together, wire together [1].

For many years, traditional studies in learning and memory have been focused on the neurobiology of memory acquisition, consolidation, and its main consequences generalization, reconsolidation, and extinction [5,6,7,8,9]. For instance, episodic memory requires two computational processes, pattern separation and pattern completion [10]. The former provides a mechanism that renders hippocampal engram cells more dissimilar for the representations of different activity patterns or similar episodes during encoding, whereas the latter plays a pivotal role in reconstructing previously established memory traces based on degraded input patterns or partial cues during retrieval [11]. Both processes involve cAMP-dependent molecular cascades that include protein kinase A (PKA), cAMP-response element-binding proteins (CREB), CCAAT enhancer-binding protein (C/EBP), c-Fos, zinc-finger protein Zif268, and other classes of transcription factors, facilitating synaptic consolidation and system consolidation processes in engram cell ensembles [12,13].

**Figure 1:**
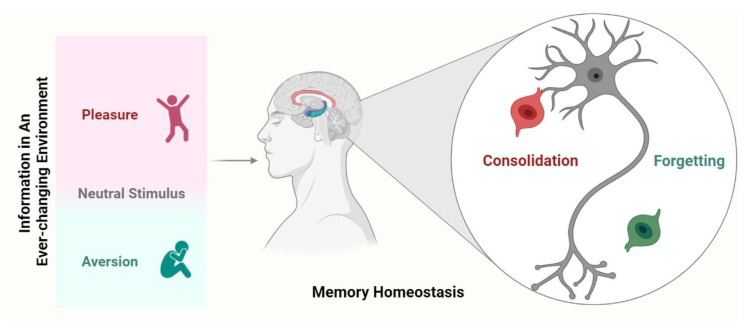
Potential mechanisms underlying memory homeostasis. Once a new memory is formed, it is mediated by both consolidation and forgetting. Consolidation allows valuable or strong memories to remain, while forgetting eliminates irrelevant (including previously well consolidated) or weak memories. Forgetting and consolidation signals continue to compete and are modulated by internal or external factors, ultimately determining the fate of the newly acquired memory.

Nevertheless, in addition to the consolidation mechanism of memory, the brain has a potential mechanism for erasing excess memory. In fact, the memory consolidation may produce harmful effects if an individual has in mind all the information from the surrounding environment. These effects may include: (1) it is unknown whether protein quality control and cellular physiology can provide the corresponding material basis for the massive memory generated and stored from every moment to the end of life; (2) if an individual remembers all the information that he/she experienced on a given day, including those that are not relevant, he/she will take nearly another day to recall it, and therefore he/she will learn nothing at all on such days; (3) if the pursuit of pleasure and aversion becomes repetitive and uncontrolled, it will cause maladaptive responses such as drug addiction or persecution maniaparanoia, respectively. In contrast, “memory erasure” indeed produces beneficial effects that an individual keeps only the information that is actually relevant and removes those that are not or become unused (see Figure 1). From this point of view, it may represent a mechanism or series of mechanisms that we call “active forgetting”. Of note, this review focuses on the intrinsic forgetting process rather than other types of passive or active forgetting, nor amnesia in any way (see below) [4,14,15].

During the last twelve decades, a great body of pioneering studies has accumulated regarding the indirect or direct evidence of intrinsic forgetting and its potential molecular, cellular, and circuit mechanisms [4,15,16,17,18,19]. Ras-related C3 botulinum toxin substrate 1 (Rac1), a member of the Rho-GTPase family, is considered to be a key controller of intrinsic forgetting. An induction of Rac1 activity triggers the decay of different forms of memory or initiation of intrinsic forgetting, whereas an inhibition of Rac1 activity enhances the persistence of memory storage or reverses intrinsic forgetting [16,20,21,22]. Cofilin, a downstream target of Rac1 activity, is a major performer of intrinsic forgetting by regulating the cytoskeleton rearrangements and actin dynamics [23]. The scaffolding protein Scribble, which interacts with Rac1 and cofilin, also is necessary for the orchestration of intracellular signaling for intrinsic forgetting [19]. This Rac1-cofilin molecular cascade results in the achievable degradation of the memory trace at the molecular, cellular, and circuit levels of engram cells, ultimately rebalancing memory consolidation.

Then again, a recent study investigated that the alteration of intrinsic forgetting may participate in the pathological mechanism of many brain diseases that are characterized by memory deficits or cognitive impairment, such as Alzheimer’s disease (AD) and autistic symptoms [24,25]. In this sense, the drugs designated to target these molecular or signaling pathways may rescue memory loss and renew the understanding of these brain disorders.

Here, we begin by briefly describing the biological and non-biological arguments supporting the existence of intrinsic forgetting in the brain and our collective ability to better understand the role of Rac1. Then, we will discuss the current evidence about memory-related neurological and psychological diseases from an intrinsic forgetting point of view.

## 2. Intrinsic Forgetting Is an Inherent Biological Mechanism for Erasing Brain Memories

### 2.1. The Theory of Forgetting in Experimental Psychology

Forgetting refers to a natural process of inability to retrieve previously learned experiences or to reuse that memory. It may occur from the beginning of memory generation; the two complement each other. Albeit experimental psychologists have been studying forgetting for a long time, the mechanism by which it occurs remains highly debated [15,26,27,28].

Typically, there are two mainstream views on forgetting: interference theory and decay theory. Interference theory postulates that forgetting is caused by inference from different stages of memory processing, such as proactive interference (prior learning), retroactive interference (subsequent learning), and retrieval interference [29,30,31,32]. For instance, a new learning presented after the previously learning event (retroactive interference) is responsible for the amnesic effect, which is mainly due to the utilization of hippocampal resources available to consolidate the original memory trace [26]. Decay theory postulates that forgetting is due to the passive or active deterioration of the memory traces due to natural molecular turnover or metabolic processes (natural decay) [33]. For example, the working memory performance falters as the length of a retention phase between a learning phase and a retrieval phase increases. Longer retention duration should result in more natural decay and, thus, more forgetting, for which the passage of time itself is not the main culprit [28].

Notwithstanding, the current research needs to solve at least several problems. (1) First, the majority of psychological studies on both theories have focused on models that restrict the field to merely enumerating the possible contributing factors. They lack deep intersection and integration with neuroscience, psychopharmacology, and cognitive sciences. Even some psychological viewpoints on forgetting argue that the brain does not have the capacity to actively or passively degrade the organic substrates of memory [4]. (2) Achieving mental quietude or ruling out interference in a retention phase (natural decay process may start) is not easy, so procedures minimizing interference differences or a new learning in the intervals may interfere with the prior learning. (3) A general unsolved question is: What is the basic unit (or fundamental property or variable) of forgetting (or memory)? Exactly what attribute in the memory traces determines forgetting in the psychological sense, or more concretely, what is processed and counted in the forgetting perception process? How do we precisely define these abstract and invariant attributes? The study of “perceptual object” and “chunk” in cognitive neuroscience may be two relatively good examples [34,35]. (4) It is self-evident that some experiment paradigms and stimulus configurations lead to findings favoring the interference hypothesis while others do not [28]. Ingenious paradigms and suited analysis methods may resolve these conflicting findings. (5) The forgetting methodology, including objective cognitive tests (or behavior monitoring) and brain imaging techniques, is limited by the insufficient precision of a single approach.

Another prominent view of forgetting is that forgetting can be classified into two major classes: passive forgetting and active forgetting [4]. Passive forgetting, triggered by a passive involvement of external or internal contributing factors, comprises the natural decay of memory traces, loss of context cues leading to difficulty retrieving memories, and retrieval interference (accumulated similar memories impede retrieval) [26,33,36]. Active forgetting, induced by active mechanisms, is composed of motivated forgetting (cognitive mechanisms or emotional motive voluntarily weaken memory traces), interference-based forgetting (proactive interference and retroactive interference), retrieval-induced forgetting (recalling some aspects of a memory inhibits the recollection of other aspects) and intrinsic forgetting [16,37,38,39].

### 2.2. The Biological and Non-Biological Evidence of Intrinsic Forgetting

Although the above-described studies have provided many different ideas or models for forgetting, only intrinsic forgetting is an elegant example of the forgetting mechanism from a neuroscience or biology perspective. Accumulated evidence has indicated that intrinsic forgetting is an inherent active forgetting process through the probable biological erosion of memory traces, such as the degradation of molecular and cellular traces and the unwiring of engram cell circuits or connections for a given memory [4,15,16,17,18,19]. This type of active forgetting likely disturbs the completeness of the memory engram or suppresses its accessibility. Unfortunately, we note that intrinsic forgetting currently is unrecognized in the experimental psychology literature. Below, we briefly describe the biological and non-biological evidence supporting intrinsic forgetting before describing the current study.

#### 2.2.1. The Non-Biological Viewpoint of Intrinsic Forgetting

Firstly, the brain storage capacity for memory may have spawned intrinsic active forgetting mechanisms [40]. Biological experimental evidence agrees with computational theories that multiple patterns (or memories) are stored in the same neural network by associative modification of the synaptic weight (or the strength of the synaptic wiring/connection) between neurons. Any unique partial input (or subset) of the target patterns (or memories) can elicit the retrieval of intact versions of previously stored patterns (or memories) by pattern completion and plausible filtering mechanisms (inhibitory interneurons) [41,42]. Nonetheless, it raises the systemic question of whether there is a constraint on the storage capacity of neural networks. If limiting, the more patterns (or memories) there are stored in the network or the more overlap there is between the stored patterns (memories), the more likely the false accessibility of engrams (or the spurious reactivation of engram cells) that do not belong to the original pattern (or target memories) [40]. That is, brain networks will soon run into the saturation problem, which may lead to memory impairments along with the passage of time [43].

Up to now, the principle of sparse encoding of the memories may be a partial solution whereby it reduces the rate of synapse utilization and the overlap among the memories (or the patterns) that are stored [44,45,46]. Despite this, it may just be a complementary mechanism, and it would be very likely that the brain has an inherent active mechanism to remove old or irrelevant memories, leading to intrinsic forgetting [4,47]. This mechanism satisfies the requirement of storage capacity and retrieval efficacy for memory and can be viewed as a factory setting that chronically brings the brain system back to its default state at a low level. In addition, environmental and evolutionary fitness require individuals to reduce the recurring and unnecessary or unwanted presentation of certain past experiences through an intrinsically active forgetting mechanism [48,49]. What is more, this adaptive forgetting may be a distinguishing feature of exceptionally creative individuals, who can often move quickly from unsuccessful ideas to more successful ones, and who are adept at reducing the habitual, logical, and mental distractions inherent in their daily routines [4,50].

Additionally, the concept of homeostasis and/or feedback regulation of biological systems is also similar to the processes of forgetting and consolidation in memory. For instance, the balance between the actions of kinases and phosphatases on the phosphorylation of a specific site on a specific protein determines the fate of the corresponding cellular activities [51,52]. Collectively, the same biological logic determines that the central nervous system must be endowed with the inherent forgetting mechanisms to balance the power of memory consolidation. From this perspective, we can speculate that the brain must have corresponding independent mechanisms that involve kinase cascade signaling, transcription factors, and synaptic cytoskeleton elimination or receptor regulation, to actively remove irrelevant or unwanted memory traces.

#### 2.2.2. The Indirect Biological Evidence for Intrinsic Forgetting

For memory consolidation, protein kinases (such as PKA), transcription factors, and other components are required for the cytoskeletal dynamics and the regulation of synapse-associated receptors (such as N-methyl-D-aspartate receptors (NMDAR) and alpha-amino-3-hydroxy-5-methyl-4-isoxazolepropionic acid receptors (AMPAR)) of synaptic plasticity, ultimately achieving the biological storage of memory in engram cells [12,13,53]. Similarly, the manipulation of several synaptic receptors, protein kinases, and transcription factors caused the forgetting of remote memories. For example, the inducible, reversible, and prolonged deletion of the forebrain-specific NMDA receptor NR1 subunit, which is generated by combing the Cre/loxP-mediated recombination system with tetracycline-controlled transactivator (tTA) systems, actively accelerates the forgetting of remote fear memories but not the deficits of recall or performance capability [54]. It has also been found that the intrahippocampal infusion of the D1 dopamine receptor antagonist SCH23390 at specific post-conditioning time points can rapidly varnish long-lasting fear-based LTMs. Conversely, an intrahippocampal application of the D1 receptor agonist SKF38393 after training transforms rapidly decaying fear memories into persistent long-term memories (LTMs) [55]. Consistently, it has been demonstrated that GluA2-containing AMPA receptors (GluA2/AMPARs) removal may be upregulated by PKMζ (an atypical protein kinase C isoform) activity, which is involved in the maintenance of LTM [56]. The pharmacological blockade of GluA2/AMPARs trafficking in the dorsal hippocampus prevents the natural forgetting of long-lasting object location memories, contextual fear memories, and consolidated associative memories induced by food-reward conditioned place preference, whereas inactivating PKMζ actively elicits the selective forgetting of well-consolidated remote fear memories [57,58,59]. Beyond that, the monospecific inhibition of α-Ca2^+^/calmodulin-dependent protein kinase II (αCaMKII, a downstream molecule of the NMDA receptor signaling cascade) in the forebrain results in the forgetting of LTM, which is consistent with previous findings that *α-CaMKII* heterozygous knockout mice exhibited an impaired maintenance of LTM [60,61]. The conditional ablation of *ERK5* (a member of the MAP kinase family), type 1 and type 8 adenylyl cyclases (*AC1* and *AC8*) in mice also contribute to the instability of remote fear memory without altering the acquisition, formation, and retrieval of LTM [62,63,64,65].

Especially, the transcriptional and translational regulatory mechanisms and epigenetic mechanisms have been also shown to modulate forgetting or memory persistence (Table 1). For instance, the conditional targeted disruption of the gene encoding the cytoplasmic polyadenylation element binding protein (CPEB3, the prion-like translational regulator), which is able to activate dormant mRNAs, impairs the stability of durable hippocampus-dependent spatial memory, ultimately inducing forgetting [66]. A high-throughput reverse genetic screen has revealed that the knockout of *steryl-O-acyl transferase 1* (*Soat1*) and *integrin beta2* (*Itgbeta2*) specifically causes the forgetting of remote memories, but it does not affect the acquisition and consolidation [67]. On top of that, the cortical DNA hypermethylation of *calcineurin (CaN)* reflects a single hippocampus-dependent associative learning in the contextual fear conditioning task. The intracortical inhibition of DNA methyltransferases (DNMT), which is responsible for writing and maintaining cytosine methylation, provoked forgetting or disturbed remote memory stability [68]. In a novel object recognition task, strong training results in remote memories and increases hippocampal histone H3 acetylation [69]. An inhibition of hippocampal histone acetylase (histone acetyl transferases, HATs) after training decreases the general level of histone acetylation and induces the rapid forgetting of remote recognition memory. In contrast, histone deacetylases (HDACs) inhibition converts a given learning experience that would not normally develop into LTM into a form of memory that is now sustainable for the long term [69,70].

Likewise, transcription factor nuclear factor κB (NF-κB) inhibition in the hippocampus reduces the histone H3 acetylation levels of the *calcium/calmodulin kinase II δ* (*Camk2d*) gene at a specific NF-κB-regulatory promoter region and impairs persistent recognition memory [69]. More than that, brain-derived neurotrophic factor (BDNF) plays a key role in the formation and persistence of long-lasting LTMs through tropomyosin-related kinase B (TrkB) receptor activation, ERK1/2 phosphorylation, and the expression of immediate early genes (IEGs) *zif-268*, *arc*, and *c-fos* [71,72,73,84]. The activity inhibition of or expression blockade of any molecular steps of the BDNF signaling cascades during the late post-training leads to rapid forgetting or an instability of remote memories [62,71,72,73,84].

All told, a wealth of indirect biological evidence (along with Table 1) supports the hypothesis that the brain has been endowed with an active mechanism for intrinsic forgetting since memory emerges.

#### 2.2.3. The Direct Biological Evidence of Intrinsic Forgetting

Several pioneering studies using *Drosophila* have demonstrated that memory decline (or decay, instability, and erasure) is an inherent active, kinase cascade-dependent process in the central nervous system [16,17,18,19,85,86].

In a Pavlovian olfactory aversive conditioning paradigm, memory retention in *Drosophila* after a single-session training generally lasts for nearly one day, and it seems to consist of two temporal phases (cycloheximide-insensitive): short-term memory (STM) that decays within minutes and mid-term memory (MTM) that persists for many hours [87]. These two labile early phases can gradually transit into a longer-lasting, more stable component that lasts over one day, anesthesia-resistant memory (ARM, consolidated memory) [87,88]. After repetitive spaced training, protein-synthesis-dependent LTM (another consolidated memory form, cycloheximide-sensitive), which lasts for at least seven days, is achieved with no apparent decay [89].

The Gal4/UAS binary system can achieve the tissue-specific expression of targeted mutant genes. *Gal80^ts^* encodes the temperature-sensitive Gal80 protein, which controls the inhibition and disinhibition of Gal4-induced targeted-gene expression at the permissive temperature and restrictive temperature, respectively. Combining the Gal4/UAS binary system with *tubulin-Gal80^ts^*, Shuai et al. showed that the specific spatiotemporal expression of the dominant-negative N17 *Rac1* mutant in the Mushroom Body (MB) of *Drosophila* successfully inhibited endogenous Rac1 activity via competitively binding upstream activators. This significantly increased the liable early memory retention from two hours to more than one day after single-session training with no marked alterations in the first 30 min after training as well as in task-relevant sensorimotor responses [16]. Instead, the constitutively active V12 *Rac1* mutant persistently increased Rac1 activity owing to its abolished inherent GTPase activity and then tremendously accelerated aversive olfactory memory decay [16].

Subsequently, blocking the synaptic transmission from a subset of MB dopamine neurons for 40 min, 80 min or a longer time window after olfactory classical conditioning led to a significant enhancement in memory retention measured at 3 h. In contrast, potentiating the MB dopamine neuron activity for 5 min, 20 min or more after learning produced a part or complete abolishment (or forgetting) in labile and cold-resistant, consolidated memories (including aversive and appetitive memories) at 3 h or 6 h [17]. These effects are determined by the dopamine receptor in the mushroom body (DAMB), which is highly expressed within the mushroom body intrinsic neurons. Mutation in the *damb* gene robustly enhances memory expression (or blocks the increased memory forgetting elicited by MB dopamine neuron activity) at time points up to one day [17,90]. Interestingly, some forms of forgetting can recover short-term synaptic depression induced by aversive conditioning in the corresponding mushroom body output neuron MBON-γ1pedc>α/β. In other words, forgetting erases physiological changes (or memory traces) generated by memory encoding. The genetic inhibition of Rac1 activity dampens normal plasticity recovery [91].

Additionally, cell division cycle 42 (Cdc42), another member of the Rho-GTPase family, is activated 3 h after a single-session training (ARM decay begins), and it is inhibited after ten-session repetitive training. The inhibition of Cdc42 activity in the MB neurons yields a prolonged ARM retention (or increases the AMR persistence) at 12 and 24 h after one session of training, which is similar to that produced by ten sessions repetitive training [86,92]. Rather, elevated Cdc42 activity by genetic manipulation speeds up the ARM decay (or induces ARM forgetting) at later time points after ten-session training, which is similar to that produced by one-session training [86]. This Cdc42-dependent forgetting is independent from cycloheximide-insensitive memory formation and forgetting, ARM formation, as well as the consolidation process itself.

Remarkably, Rac1 activity inhibits the actin-depolymerizing activity of Cofilin by the sequential activation of p21-activated kinase (PAK)/LIM kinase (LIMK), which modulates activity-dependent actin cytoskeleton remodeling and synaptic plasticity, and in turn, it is responsible for intrinsic active forgetting [4,93,94,95,96]. For instance, Cofilin hyperactivation increases memory retention at 3 h similar to that observed with Rac1 inhibition [16]. A recent study has demonstrated that the scaffolding protein Scribble is part of a downstream pathway of dopaminergic signal inputs triggered by the ongoing dopamine acting on DAMB receptors. It physically interacts with Rac1, Pak3, and Cofilin in the MB neurons, together contributing to intrinsic active forgetting. For example, the genetic disruption of Scribble expression in MB neurons or dopaminergic neurons leads to forgetting impairment of normal memory [19].

On the whole, this evidence, along with Table 2, indicates that intrinsic forgetting is an inherent and separate biological mechanism for erasing unused or unwanted memories, and it is an indispensable part of the brain’s memory management system.

## 3. Molecular Mechanism and Regulation of Rac1-Dependent Intrinsic Forgetting

Congruent lines of evidence have proposed that intrinsic active forgetting may be caused by different molecular, cellular, and circuit mechanisms for erasing the distinct aspects of an established experience or/and memory of a different nature, including Rac1-dependent, Cdc42-dependent, dopamine activity-dependent, and undiscovered forgetting [4,15,16,17,18,19,98,100]. For example, Rac1-dependent intrinsic forgetting induces an active rapid removal of the liable early memory, whereas Cdc42-dependent intrinsic forgetting accelerates remote memory decay [16,20,86,101]. Interestingly, behavioral state-induced dopamine neuron activity, which is mediated by dopamine 1-type receptors, is required for memory formation or acquisition. However, the same dopamine neuron activity, which is stimulated by the DAMB receptor, also contributes to modulating memory decay or forgetting [15,17,19,102]. However, this field is still in its infancy. Here, we focus on Rac1-dependent intrinsic forgetting, which is a well-charactered and elegant example of an intrinsic forgetting mechanism.

### 3.1. The Introduction of Rac GTPase

In metazoans, the Ras superfamily of small GTP-binding proteins plays a vital role in cellular signaling transduction and organization during development and adulthood. According to the diversification of sequence, structure and function, the Ras superfamily is divided into five smaller subfamilies: Rho, Ras, Rab, Ran, and Arf [105]. Rac GTPase (~21 kDa) is a member of the Rho subfamily, which consists of almost 20 members. They are mainly involved in gene expression and actin organization (and/or the regulation of microtubule cytoskeletons), which determine the morphogenesis (or development and morphology) and remodeling of dendritic spines, dendrites, and synapses. The Rac GTPase family has four members: Rac1, Rac2, Rac3, and RhoG [92,106]. Rac GTPases are activated by guanosine nucleotide exchange factors (GEFs) with a GTP-bound state and are inactivated by GTPases activating proteins (GAPs) with a GDP-bound state [107]. Rac GTPase activity is negatively controlled by guanine nucleotide dissociation inhibitors (GDIs) via the sequestration of inactive Rac GTPases in the cytoplasm [108]. This cycle is presumably regulated by these GTPase effectors through the translocation to membranes, relief of auto-inhibitory intramolecular interactions, and conformational change [109].

Characteristically, GEFs promote GDP dissociation from inactive Rac GTPase and the conversion from a GDP-bound low-affinity complex into a GDP-free/GEFs high-affinity complex by conformational change. This GEFs high-affinity complex preferentially binds GTP available in excess over GDP during the cellular microenvironment [110]. By contrast, GAPs accelerate the intrinsic hydrolysis activity of the Rac GTPases by several orders of magnitude, promoting the hydrolysis of GTP to GDP and the conversion of the GTPases from being constitutively active to an inactive state [111]. Notably, the interaction between GAPs/GEFs and Rac GTPases in this cycle is required to bind on the cell surface via a prenylation post-translational modification [112].

### 3.2. The Structure and Effector Signaling of Rac1

**Figure 2:**
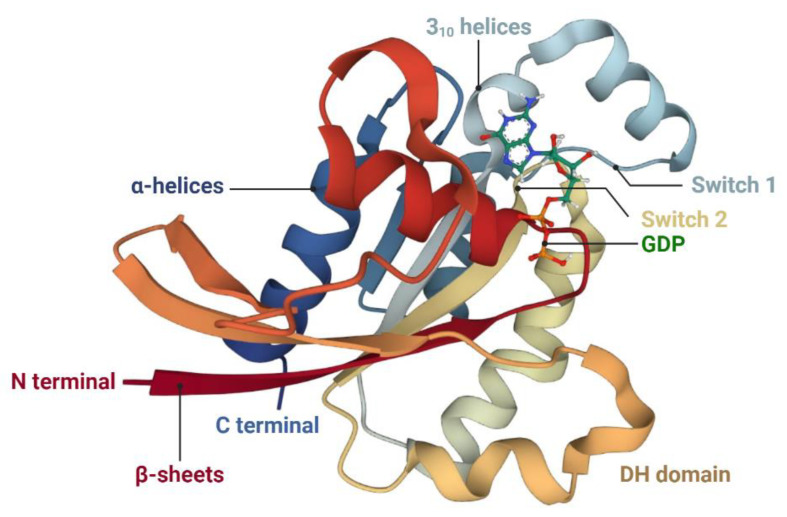
Schematic representations of the tertiary structure of human Rac1 in the low-affinity state. Rac1 consists of one central β-sheet with six strands, six α-helices, and two short 3_10_ helices. The structure is obtained from the RCSB Protein Data Bank (RCSB PDB) (Toyama et al., 2019 [115]). Abbreviations: DH domain, Dbl-homology domain.

Rac1 is the best-known and well-characterized member of the Rac GTPases, which usually affect the different effectors or signaling cascades, and eventually, mediate actin dynamics [113,114]. The Rac1 protein structure is composed of six α-helices, two short 3_10_ helices, and a central β-sheet with six strands [115] (see Figure 2). The five strands in this β-sheet are parallel to each other, and the sixth strand is antiparallel to the others [116]. Structurally, Rac1 consists of an insertion domain, which extends from leucine (Leu) 117 to isoleucine (Ile) 137, and a nucleotide-binding pocket, which encompasses several highly conserved loops, such as ^10^GDGAVGKT and ^57^DTAG phosphate-binding loops, ^115^TKLD and ^158^SAL guanine recognition loops, and a less conserved effect loop made up of residues 30–40 [116,117]. The insertion domain that presents a highly charged surface is exposed and mobile, and it displays a loosely packed hydrophobic core, which is formed by residues valine (Val) 85, L-threonine (Thr) 125, Ile 126, Leu 129, and proline (Pro) 136. The effect loop plays a key role in the hydrolysis of GTP and binding of GAPs as well as downstream signal transduction [116,118].

As seen with all other Rac GTPases, Rac1 switches between GDP-bound low-affinity and GTP-bound high-affinity states via the catalytic action of GAPs and GEFs. Along with this prominent role in state cycles, Rac1 also changes between the cytosol and membrane-bound forms through post-translational modification, which is essential for translocation to membranes [112,119].

**Figure 3:**
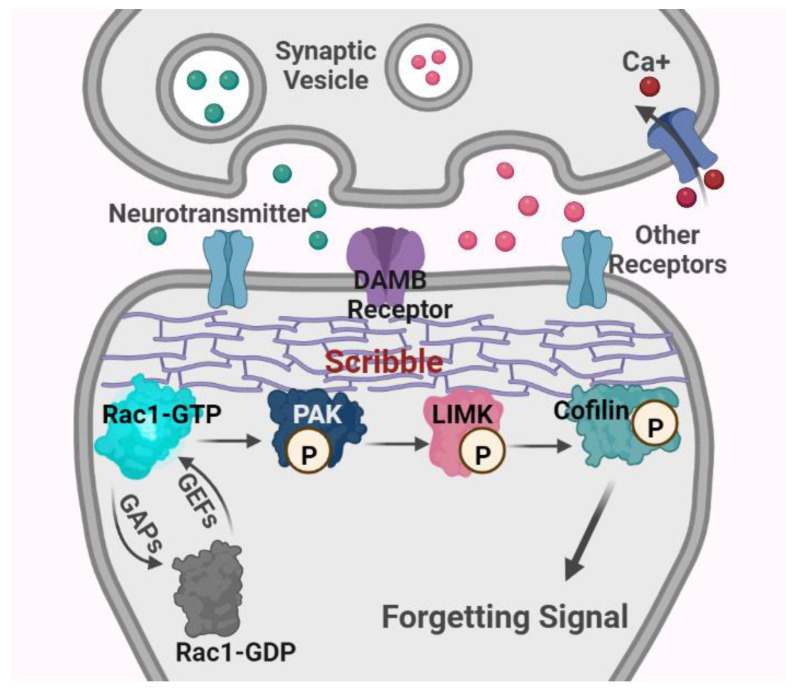
Schematic diagram of major mechanisms of Rac1-dependent intrinsic active forgetting in *Drosophila*. Abbreviations: Rac1, Ras-related C3 botulinum toxin substrate 1; GTP, guanosine triphosphate; GDP, guanosine diphosphate; GEFs, guanosine nucleotide exchange factors; GAPs, GTPases activating proteins; PAK, p21-activated kinase; LIMK, Lin11, Isl-1 and Mec-3 kinase; DAMB receptor, the dopamine receptor in the mushroom body; P, phosphorylation.

Active Rac1 binds to and phosphorylates its downstream target, Pak, through the Rac-binding domain of Pak, which localizes to regions of actin cytoskeletal assembly [120,121]. Concomitantly, active Pak activates LIMK through the phosphorylation of threonine 508 within LIMK’s activation loop. This catalysis process requires the substrate determinants within LIMK’s activation loop (a highly basic 11-amino-acid insertion from arginine 495 to arginine 506) and structural determinants in both the nitrogen-terminal regulatory (including the auto-inhibitory regulatory domain, amino acids 83–149, and the p21-binding domain, amino acids 67–86) and the carboxy-terminal catalytic domains (amino acids 232–544) of Pak [95]. Activated LIMK then phosphorylates the actin-regulatory protein cofilin at an N-terminal serine residue 3, thereby inhibiting its F-actin depolymerization activity and resulting in the accumulation of actin filaments [94,122]. The specific inhibition of Pak activity in its auto-inhibitory domain blocks LIMK-mediated actin cytoskeletal changes, and the inactivation of LIMK disturbs Rac1-dependent and Pak-dependent actin dynamics [95] (see Figure 3).

### 3.3. The Rac1-Mediated Biological Function in the Brain

A fundamental property of the dynamics and organization of the actin cytoskeleton is the ability to underlie the formation, maturation, maintenance, strength and plasticity of synaptic connections. The Rac1-cofilin signaling cascade therefore serves as a critical control pathway for cytoskeletal dynamics in the spine morphology, dendritic spine and synaptic plasticity. For example, hippocampal neurons in *LIMK-1* knockout mice showed a significant alteration in cofilin phosphorylation, abnormal distribution of filamentous actin with a low level of actin filaments at mature synapses, and functional impairment in hippocampal LTP. Consistent with synapse deficits, knockout mice also exhibited apparent abnormalities in behavioral performances, such as impaired spatial learning and fear conditioning [123]. The conditional deletion of *n-cofilin* (a non-muscle cofilin) in the forebrain of mice (n-cof^flx/flx, CaMKII-cre^) specifically increased the F/G-actin ratio in the synaptic compartment, which was accompanied by increased synapse density and an enlargement of dendritic spines. Apart from affecting spine morphology, a loss of n-cofilin-dependent synaptic actin dynamics in the hippocampus specifically altered AMPAR availability and synaptic plasticity (LTP and LTD), ultimately leading to severe behavioral impairment in all types of associative learning tasks [124]. In addition, the protein Scribble is a highly conserved cell polarity regulator, which contains an amino-terminal Leucine-Rich Repeats region and four PSD-95/Discs-large/ZO-1 domains. It acts as a scaffold to assemble and position diverse protein complexes at the plasma membrane, regulating planar polarity, adhesion, oriented cell division, directed cell migration, and synaptogenesis [125,126,127,128,129]. Scribble mutation results in defects in convergent extension and apical constriction (polarized cell intercalation) during mammalian neural tube closure, such as the failure of both cell apical constriction and cell wedging [130,131]. *Scribble* mutant embryos in mice exhibit abnormal expression of the junctional proteins Par3, Par6, ZO-1, E- and N-cadherins, and the cytoskeletal proteins actin and myosin [132]. The conditional deletion of *Scribble* in the mouse telencephalon results in partial corpus callosum and hippocampal commissure agenesis, cortical thickness reduction (microcephaly), and accompanying psychomotor deficits, including locomotor activity impairment and memory alterations [133].

Notably, the manipulation of Rac1 activity also leads to changes in the neuronal actin cytoskeleton, dendritic spine morphogenesis, synaptic plasticity and additional neuronal functions [20,134,135,136,137,138]. For instance, hippocampal mature pyramidal neurons expressing dominant-negative *Rac1* cause a progressive elimination of dendritic spines [134]. The selective ablation of *Rac1* in the forebrain excitatory neurons in vivo not only impairs the synapse structure, including an increased spine head size, mean PSD length, synaptic cleft widths and reduced number of PSD-95^+^ clusters, but also affects synaptic plasticity with consequent defects in working/episodic-like memory and hippocampus-dependent spatial learning [136]. The overexpression of a dominant-negative *Rac1* in hippocampal pyramidal neurons also shows a similar phenotype, such as decreased spine density, synaptic motility and longer and thin filopodia-like spine [134,139]. The inhibition of Rac1 activity by an inhibitor EHT 1864 effectively blocks AMPAR transmission and structural enlargement of the dendritic spine (structural LTP or sLTP) [140].

In contrast, Purkinje neurons and pyramidal neurons expressing constitutively active Rac1 results in an increased spine number and decreased spine size [135,141]. The specific activation of Rac1 by a novel synaptic optoprobe, activated synapse targeting photoactivatable Rac1 (AS-PaRac1), in recent potentiated spines elicits the particular shrinkage of AS-PaRac1-containing spines and disrupts a newly acquired motor learning [3]. The overexpression of constitutively active Rac1 and wild-type Rac1 in cultured hippocampal neurons induces a significant increase in the size of pre-existing spines and the amount of AMPAR clusters, promoting an increase in the amplitude of miniature EPSCs (mEPSCs) [142]. Moreover, Rac1 also affects the neuronal actin cytoskeleton through recruiting the cyclin-dependent kinase (Cdk5), which directly phosphorylates the WASP-family verprolin homologous (WAVE1) protein on the three serine residues [143]. The WAVE protein consequently activates the actin-related protein 2/3 (Arp2/3) complex that concentrates in dendritic spines and acts upon the sides of pre-existing filaments, contributing to the formation of dendritic spines [144].

Along with this prominent role in spine morphogenesis and function, Rac1 is implicated in learning-evoked neurogenesis in the adult mouse hippocampus and axonal morphogenesis in *Drosophila* mushroom body neurons [137,145]. Rac1 is also involved in alterations in synaptic efficacy, such as the dynamic control of AMPA receptor functions and localization [142,146,147]. Taken together, these results suggest that Rac1 signaling cascades are intimately involved in regulating dynamic actin cytoskeleton, synaptic structure and function.

In the context of these mechanisms, the concepts and mechanisms of Rac1-dependent intrinsic forgetting proposed by Zhong and colleagues can be well understood [4,16]. Several studies have validated that Rac1 is a major participant in intrinsic forgetting, and the regulation of the upstream (DAMB, the Scribble protein) and downstream (cofilin) of Rac1 also alters the process of intrinsic active forgetting [16,17,19,20,22] (see Figure 3).

### 3.4. Rac1-Dependent Intrinsic Forgetting Is an Evolutionarily Conserved Mechanism in Multiple Species

In *Drosophila*, constitutively active Rac1 in the MB neurons accelerates memory decay in Pavlovian olfactory aversive conditioning, whereas constitutively negative Rac1 inhibits intrinsic forgetting [16]. The overexpression of constitutively active Rac1 that is unable to bind PAK fails to induce intrinsic memory forgetting [19]. When the DAMB receptor is genetically reduced, odor conditioning-induced memory expression is dramatically enhanced a few hours afterward. However, the reversal odor conditioning-mediated memory expression is obviously disturbed, indicating that *damb* mutant flies are significantly defective in their ability to actively forget the first contingency [17]. Genetic deletion of the Scribble protein has a similar effect on memory decay to *damb* mutation. Constitutively active cofilin also increases memory retention after conditioning, which is consistent with its role in intrinsic active forgetting [16] (see Figure 3).

In mice, enhancing basal Rac1 activity inhibits the acquisition of non-spatial visual-discrimination memory in the object recognition task and spatial memory in the MWM task [148]. Selectively ablating Rac1 activity in adult pyramidal neurons impairs hippocampal LTP and the ability to acquire spatial memory on several training days on the MWM task without affecting normal memory evaluated subsequently in probe trials [136]. The inhibition of Rac1 activity resulting from the mutation of *ArhGAP15* (coding for a Rac-specific GAP protein) reduces the freezing response during auditory trace fear conditioning [149]. The manipulation of hippocampal Rac1 activity using adeno-associated viruses (AAVs) carrying dominant-negative Rac1 increases the retention of object recognition-induced memory that commonly lasts less than 72 h, while elevated Rac1 activity using AAVs carrying constitutively active Rac1 accelerates memory decay to less than 24 h [20]. Social recognition memory, contextual fear memory, and cocaine-associated memory performance also show a similar pattern to that produced by the aforementioned research through viral-mediated regulation strategies of Rac1 activity [22,23,150,151,152].

In rat, the spaced training of contextual fear conditioning results in the inhibition of hippocampal Rac1 activity and heightened contextual fear. The pharmacological inhibition of Rac1 activity in the hippocampus enhances fear memory in massed training, whereas the intrahippocampal injection of Rac1 activator impairs contextual fear memory in spaced training [21]. Massed extinction training activates hippocampal Rac1 and leads to a long-term extinction of fear memory. The Rac1 inhibitor prevents fear extinction in massed extinction training, while enhanced Rac1 activity boosts the extinction of contextual fear memory in long-spaced extinction training [153]. The extinction of aversive memory in naloxone-precipitated opiate withdrawal-induced conditioned place aversion (CPA) upregulates Rac1 activity in the ventromedial prefrontal cortex (vmPFC). The disruption of Rac1 protein expression within the vmPFC suppresses CPA extinction, and the expression of constitutively active Rac1 accelerates CPA extinction [154].

In humans, AD patients exhibit a considerable upregulation of Rac1 levels compared to an age-matched control [155,156]. The tree shrew (*Tupaia belangeri chinensis*), a close relative of nonhuman primates, has a similar time of day-dependent pattern of hippocampal Rac1 activity to rats [157,158]. Unfortunately, these studies in human beings and non-human primates do not include clear experiments to determine whether Rac1-dependent intrinsic forgetting participates in the brain’s memory management system. Altogether, the current evidence further supports the observations that Rac1-dependent intrinsic active forgetting is an evolutionally conserved mechanism for memory erasure.

### 3.5. The Regulation of Rac1-Dependent Intrinsic Forgetting

Hitherto, evidence exists that internal states and external factors may promote or prevent Rac1-dependent intrinsic forgetting, such as activity, sleep, stress, emotion, and others [4,18,22,151,158,159]. It is well established that hippocampal ensemble memories (or newly acquired information) are reactivated and enhanced by sleep after post-encoding across species [160,161,162]. One accepted mechanism is that rapid eye movement (REM) sleep and slow-wave sleep (SWS) promote synaptic consolidation and system consolidation, respectively, contributing to memory enhancement or changes of memory representations during sleep [161,162]. Another possibility is that the sleep state may diminish or inhibit intrinsic active forgetting. For example, Jiang et al. identified that hippocampal Rac1 activity in nocturnal rats undergoes a time of circadian rhythm-dependent alteration, and a more stable fear memory can be acquired from lower points of Rac1 activation during the night [158]. Berry et al. suggested that sleep after training facilitates fear memory retention, and decreases ongoing dopaminergic activity-based (Rac1 activity is downstream of MB neuron signaling) forgetting in *Drosophila* [18]. On the contrary, arousal increases robustly ongoing dopaminergic activity with locomotor activity and accelerates forgetting [18]. Stressful events (such as foot shocks and exogenous corticosterone treatment) induce the downregulation of hippocampal Rac1 activity and impair fear memory or reduce fear memory expression [159].

Furthermore, acute social reward-evoked emotions are sufficient to suppress protein synthesis inhibitor-induced hyperactive Rac1, which is followed by rescued effects on amnesic memory, while acute social stress-evoked emotions can increase hippocampal Rac1 activity (which is normally the hypoactive state after fear conditioning) with reduced fear memory expression [151].

## 4. Rac1-Dependent Intrinsic Forgetting in Brain Disorders

Many central nervous system diseases are characterized clinically by cognitive impairment, which is involved in common pathophysiology in synaptic structure and function, i.e., AD, major depressive disorder (MDD), Huntington’s disease (HD), autism spectrum disorder (ASD), fragile X Syndrome (FXS), and post-traumatic stress disorder (PTSD), to name a few [163,164,165,166,167,168,169]. Taking into consideration the core function of Rac1 in synaptic actin dynamics, cytoskeleton rearrangements and memory management, it is not surprising that Rac1-dependent intrinsic forgetting may play a fundamental (or partial) role in memory deficits among several brain diseases. Here, we only discuss the relationship between Rac1-dependent intrinsic forgetting and these brain disorders in this perspective, not other aspects of the disease.

### 4.1. AD

Despite continuing debate about AD pathogenesis, it is well accepted that the typical pathological characteristics of AD include progressive amyloid-beta (Aβ) deposition, the accumulation of neurofibrillary tangles, and tau protein hyperphosphorylation, accompanied by synaptopathology and cognitive decline [163,170]. One developmental neuroscience study found that neuronal Cdc42/Rac1 is upregulated in AD brains in comparison to age-matched controls, and it exhibits significant overlap with early actin cytoskeletal abnormalities [171]. Subsequently, several studies have demonstrated that hyperactive Rac1 is intimately associated with Aβ precursor protein (APP) processing and actin polymerization that causes memory deficits with age in AD [172,173,174,175,176]. The fibrillar Aβ_1–42_ peptide in cultured hippocampal neurons promotes membrane translocation and the activation of Rac1 as well as the increase in lamellipodia and filopodia formation through a Tiam1 (a Rac1 GEFs)-dependent mechanism, whereas Rac1 inactivation through transfecting dominant-negative forms inhibits the abnormal enhancement of actin polymerization [176]. The pharmacological inhibition of Rac1 activity by an inhibitor EHT 1864 blocks or reduces Aβ_40_ and Aβ_42_ production in cell lines endogenously expressing wild-type and human mutant APP and in Hartley albino guinea pigs (an AD model for physiological APP processing and Aβ generation), respectively [175]. Recently, there has been growing evidence suggesting that Rac1 activity is aberrantly elevated in AD patients and various AD animal models, such as *APP/PS1* mice, *ApoE*^-/-^mice, *3xTg-*AD mice, AlCl3 and D-gal co-induced AD rat, and human Aβ_42_ transgenic fly [25,155,177,178,179,180,181,182,183,184]. For instance, nucleus basalis neurons of the cholinergic basal forebrain (CBF) in AD patients exhibit significant hyperactive Rac1 that colocalizes with tau cytoskeletal markers [177]. Rac1 activity in the hippocampus of AD patients is also upregulated without affecting total Rac1 expression in comparison to healthy age-matched controls, which is consistent with the observation in the hippocampus tissues of *APP/PS1* mice and in the whole brain of Aβ_42_ transgenic fly [25].

Accordingly, the intragastric administration of the Rac1 inhibitor EHop-016 can rescue Rac1 hyperactivation and LTP fast decay in the hippocampus regions and then ameliorate accelerated memory decay in the old *APP/PS1* mice during the MWM task. Similarly, the genetic manipulation of hippocampal Rac1 activity through injecting recombinant AAV-CaMKIIα-Rac1-dominant negative viruses alleviates memory deficits in the MWM paradigm without influencing learning or memory formation. From the perspective of intrinsic forgetting, these studies indicate that the observed memory defect in AD mice and the AD fly results from abnormal Rac1-dependent intrinsic forgetting [16,20,25].

Interestingly, Borin et al. showed that Rac1 levels are significantly lower in the frontal cortex of human AD samples and 7-month-old *3xTg*-AD mice compared to the healthy controls. Meanwhile, the application of different constitutively active Rac1 peptides in primary cortical neurons elicits an accumulation of Aβ precursor protein processing and induces the translocation of nuclear oncogene SET as well as an increase in the tau hyperphosphorylation at residue pT181 [155]. Moreover, hippocampal Rac1 is activated in 6-week-old *3xTg*-AD mice and total protein levels are reduced at 7 months, but the intranasal administration of constitutively active Rac1 peptides in 6.5-months-*3xTg*-AD mice inhibits dendritic spine loss and rescues spine and synaptic abnormalities [155]. This apparent paradox may be owing to a possible dual role of Rac1 activity across the different stages of the pathogenesis or heterogeneous response of brain region in AD, or it could be caused by other factors.

### 4.2. MDD

The rapid learning of stubborn memories and/or adaptive or maladaptive coping strategies (such as learned helplessness and immobility) may result in the enduring biased negative emotional processing for an established memory. This is the universal state in MDD patients suffering from the psychological and physical effects produced by uncontrollable or controllable stress exposure [185,186,187,188]. In line with this direction, it is possible to speculate whether long-lasting old unfriendly memories in MDD are involved in abnormal intrinsic active forgetting. Transcriptional profiling in chronic social defeat stress-induced depression mice reveals a marked downregulation of Rac1 levels in the NAc, which are correlated with enduring social avoidance behavior [189]. This transcriptional reduction is controlled by reduced permissive histone 3 acetylation (acH3) within the promoter region of *Rac1* and upstream regulatory regions, which is followed by an enhanced repressive histone H3 lysine 27 (H3K27me3) methylation in upstream regulatory regions of the *Rac1* promoter [189,190]. The intra-NAc minipump infusion of histone deacetylases (HDACs) inhibitor (MS-275) normalizes Rac1 mRNA levels and rescues social defeat-induced social avoidance. Furthermore, knockout of the *Rac1* gene and the overexpression of dominant negative Rac1 through herpes-simplex virus-mediated manipulation robustly induces susceptibility to depression-related phenotypes in mice, such as anhedonia and social avoidance. Conversely, the viruses-mediated overexpression of constitutively active Rac1 after chronic social defeat reverses depression-related behavior with a restoration of Rac1 levels [189]. Repeated exposure to chronic unpredictable mild stress (CUMS, a valid depression paradigm) downregulates the expression and active form of hippocampal Rac1 in the rat. The administration of S-Ketamine, a rapid and long-lasting antidepressant drug, reverses the reductions in expression and activity of hippocampal Rac1 and rescues CUMS-evoked LTP damage and depression-like behavior. The inhibition of Rac1 activity using the inhibitor NSC23766 is able to counteract ketamine-mediated molecular alteration in the hippocampus and antidepressant effects [191]. From the perspective of regulation of Rac1 activity, these studies are in line with the concept and methodology of intrinsic forgetting.

Consistent with the observation in the rodent models, Rac1 mRNA expression in the post-mortem NAc of MDD patients is significantly downregulated and is mediated by decreased acetylation within the promoter region of *Rac1* and upstream sequence as well as increased methylation upstream of the transcription start site of *Rac1* [189]. Expression levels of *PAK1* in the PFC and *PAK3* in the hippocampus are also abnormally downregulated in subjects with depression in comparison to controls [192]. In addition, the breakpoint cluster region (*BCR*) gene encoding a GAP, the family of dedicator of cytokinesis (*Dock*) 9 encoding a GEF, the Gem-interacting protein (*GMIP*) gene encoding a novel GAP, and the triple functional domain (TRIO) protein (a GEF for Rac1) is associated with bipolar disorder or MDD in subjects [193,194,195,196,197]. Rac1 regulatory molecular is also involved in depression-like behaviors of animal models, such as α2-chimaerin (a GAP) and WfS1 [198,199]. In brief, these studies suggest that abnormal Rac1-dependent intrinsic forgetting may be involved in depression-related phenotypes in mammalian animals, including humans.

### 4.3. HD

HD is a fully penetrant, progressive neurodegenerative disease with an extensive spectrum of clinical symptoms and signs, such as incoordination, cognitive decline, chorea and dystonia, and behavioral difficulties [200]. A recent study indicated that Rac1 activity is significantly elevated in the striatum of *HD Q140/Q140* mice and in cultured primary cortical neurons of HD-mutant mice [201]. Kalirin-7 (Kal7), a GEF for Rho-like small GTPases, is downregulated in the cortex of *R6/1* mutant transgenic mice (HD mice model) and in the cerebral cortex and putamen of HD patients. Simultaneously, the reduction in Rac1 activity caused by decreased Kal7 is also found in the cortex of *Hdh^Q7/Q111^* knock-in mice [202]. Notably, a large-scale Huntingtin protein interaction network suggested that dysregulation in Rac1 is involved in the pathological mechanisms in HD [203]. Thus, these studies may imply that Rac1-dependent intrinsic forgetting may be closely related to the pathogenesis of HD.

### 4.4. ASD

ASD is a highly prevalent, neurodevelopmental disorder in children with a core phenotype, such as age-inappropriate deficits in social communication and interaction as well as restricted and stereotypic behavior [204]. Far-reaching efforts have been devoted to discerning the convergence point of the targets, pathways, and functions of the ASD-risk genes to promote mapping out apparent causal paths. A computational network model for gene–environment interactions (GENVI model) in the ASD context showed that Rac1 has a strong etiological relevance in autism-related neuropathological events [205]. Dock4, an atypical GEF for Rac1, is critical for ASD, whereby the microdeletion of a *Dock4* mutant results in defective neurite growth and synaptic connections via activation of Rac1 [206]. *Dock4* knockout mice intriguingly exhibit a series of ASD-like behaviors, containing impaired social interaction, unnatural isolation-induced pup vocalizations, abnormal object and spatial learning, and elevated anxiety-like behavior, which is consistent with that of the conditional ablation of *Dock4* in hippocampal CA1. Dock4 deficiency causes a marked reduction in hippocampal Rac1 activity, while hippocampal Rac1 replenishment in the *Dock4* KO mice reverses autism-like social impairments in these mice [207,208]. Growing evidence suggests that cytoplasmic autism susceptibility candidate 2 (AUTS2) plays a regulatory role of Rac1 in the pathology of ASD [209,210]. For instance, *Auts2* heterozygous mice display significant ASD-like behaviors. Genetic deletion of the *Auts2* gene leads to defects in neuronal neuritogenesis and migration through the inactivation of the Rac1 pathway in mice, and it induces a battery of behavioral deficits, including impaired auditory fear conditioning memory and novel object recognition capability [210,211].

Remarkably, the most straightforward demonstration consistent with the Rac1 regulatory factor is that the disruption of Rac1 at glutamatergic synapses contributes to ASD-like behaviors in ASD animal models [205,212,213]. The *de novo* missense mutation in Rac1, which inhibits Rac1 activation by obstructing Rac1′s GTP-binding pocket, impairs the LTP and cognitive function [213]. The inhibition of Rac1 activity induces social interaction deficits in wild-type mice, whereas activating Rac1 can rescue autism-like defects in Shank3-deficient mice (ASD model) [212]. Dong et al. proved that Rac1-dependent forgetting is a functional converging point for multiple autism-risk genes [24]. These evidences suggest that Rac1-dependent intrinsic forgetting is involved in ASD. Moreover, ArgGAP32 (a GAP for Rho GTPase), P-Rex1 (a specific GEF for Rac1), the GEF1 domain of Trio, and PAK, are also associated with autism-like social behavior in ASD patients and rodent models [214,215,216,217,218].

### 4.5. FXS

FXS is the common inherited cause of intellectual disability and ASD, typically resulting from the transcriptional silencing of *fragile X mental retardation type 1* (*Fmr1*) on the X chromosome and loss of the encoded protein, FMRP [219]. FXS patients often present with varying degrees of behavioral alterations, such as impulsivity, hyperactivity, mental retardation and anxiety [220]. Considerable evidence links aberrant Rac1 signaling to the pathogenesis of FXS associated with changes in the spine and synapse and memory deficits. For example, Rac1 mRNA is present in the *Fmr1*–messenger ribonucleoprotein complexes and genetically interacts with Fmr1 in *Fmr1* mutant flies, contributing to responsibility for dendritic development. Increased Rac1 expression in the DA neurons of *Fmr1* mutants can partially rescue the dendritic phenotype induced by *Fmr1* overexpression [221]. The genetic mutation of *cytoplasmic FMRP-interacting proteins CYFIP* in *Drosophila* impairs the axons and synapses, much like mutations in *Rac1* and in *Fmr1*. CYFIP and Rac1 interact biochemically and genetically with each other in response to the cross-talk between synaptic cytoskeleton remodeling and translational control [222,223]. *Fmr1* knockout mice cause an overactivation of Rac1 (or increased levels of membrane-bound Rac1) and its effector PAK1 and cofilin phosphorylation in the brain, with FXS phenotypes including hyperactivity, learning deficits and macroorchidism [224,225,226,227,228]. The pharmacological reduction in Rac1 partially reverses altered long-term plasticity in *Fmr1* knockout mice [225]. PAK1 inhibition rescues cofilin signaling and ameliorates synaptic defects, sensory processing and behavioral abnormalities in FXS mice [227,229]. The injection of constitutively active cofilin into the somatosensory cortex of FXS model mice normalizes the abnormal dendritic spine phenotype and synaptic signaling [227]. Martinez et al. also found that the inhibition of Rac1 activity in the hippocampus of *Fmr1* knockout mice improves the LTP and hippocampus-related memory in fear conditioning [230]. These pieces of evidence suggest that Rac1-dependent intrinsic forgetting may be partially involved in the pathological mechanism of FXS.

Above all, Rac1 activity in the brain may play a critical role in neuronal morphological abnormalities and memory-related brain disorders. We owe it to the patients to change our minds to ponder other possibilities beyond the known pathological mechanisms and potential therapies for memory-related brain disorders, such as Rac1-dependent intrinsic forgetting [4,16]. For instance, Dong et al. have reported whether ASD risk genes impair Rac1-dependent active forgetting [24]. This represents an underexplored research avenue for dissecting the pathogenesis of brain diseases and designating drug candidates. However, elucidating the neural mechanisms of Rac1 intrinsic active forgetting and its potential role in memory-related neurological and psychological diseases is still challenging.

## 5. Conclusions

Every day, animals, as well as human beings, constantly receive a plethora of stimuli from the surrounding environment, but just a tiny fraction of them will become memories. Or, put differently, memories are islands in an ocean of forgetting over time. Apart from other forms of forgetting from experimental psychology (such as interference-based forgetting, retrieval-induced forgetting and motivated forgetting, etc.), the brain has an inherent and separate biological mechanism that corresponds actively to the removal of unwanted or unused memories, namely, intrinsic active forgetting. This, along with acquisition, consolidation, generalization, reconsolidation and extinction, constitute the necessary conditions for brain memory management. As we have discussed, Rac1-dependent intrinsic forgetting is a well-charactered and elegant example of the forgetting mechanism from a neuroscience perspective. Classically, Rac1 was known as the control center of actin dynamics, affecting spine morphology, dendritic spine and synaptic plasticity through the different effectors or signaling cascades (such as Pak, LIMK, and Cofilin); nowadays, novel insight recognizes that also intrinsic active forgetting relies on this molecular and its signaling. For instance, the induction of Rac1 activity elicits forgetting processes or memory decay, whereas the inhibition of Rac1 activity enhances memory persistence or mitigates intrinsic forgetting, both without interfering in memory acquisition or formation. The findings from several other labs using different strategies to investigate multiple types of memory converge to support the perspective that Rac1-dependent intrinsic active forgetting is an evolutionally conserved mechanism across species. In a physiological condition, internal states and external factors including activity, sleep, stress, and emotion are both involved in the regulation of Rac1-dependent intrinsic forgetting. However, under pathological conditions, Rac1-dependent intrinsic forgetting may result in alterations in brain memory homeostasis that could induce memory decline or stubborn memories in memory-related neurological and psychological diseases. In line with this sense, it would be encouraging to determine whether at least some impaired memories are based on aberrant Rac1-dependent intrinsic forgetting and, if so, use the conversion of Rac1 activity to modulate forgetting as a means of characterizing and treating (or attenuating) the memory deficits in the brain diseases.

## Figures and Tables

**Table 1:** Molecular manipulation in animal models (of humans) induced active forgetting or impaired memory persistence.

Molecular Target	Manipulation	Experimental Paradigm	Behavioral Effect	References
Mouse NMDA receptor (NR1 subunit)	Inducible and reversible knockout (prolonged absence)	Fear conditioning	Forgetting remote fear memories	[54]
Rat D1 receptor	Pharmacological inactivation	One-trial, step-down, inhibitory avoidance task	Forgetting of LTMs	[55]
Rat hippocampal GluA2/AMPAR	Blocking GluA2/AMPAR removal	Fear conditioning/conditioned place preference/object location recognition	Preventing normal forgetting of LTMs	[57]
Mouse *αCaMKII*	Inducible and forebrain-specific protein knockout; *CaMKII* heterozygous knockout mice	Contextual- and cued-fear conditioning	Forgetting of LTMs	[60,61]
Mouse *ERK5*	Inducible and conditional knockout	Contextual- and cued-fear conditioning	Forgetting remote fear memories	[62]
Mouse *AC1/AC8*	Genetic knockout	Contextual fear conditioning	Impairing the stability of remote contextual fear memory	[63,64,65]
Rat PKMζ	Pharmacological inactivation by myristoylated PKMζ pseudosubstrate inhibitory peptide (ZIP)	Fear conditioning/ conditioned taste aversion	Forgetting of LTMs	[56,58]
Mouse *CPEB3*	Regional- and temporal-specific deletion	Morris water maze task (MWM)	Forgetting remote spatial memory	[66]
Mouse *Soat1/Itgβ2*	Reverse genetic mutant strains	Context fear conditioning/conditioned taste aversion	Forgetting remote memories	[67]
Mouse *CaN* methylation	DNMT inhibitor (RG108, 5-azadeoxycytidine, and zebularine)	Context fear conditioning	Forgetting remote memories	[68]
Mouse HDAC	HDAC inhibitors	Novel object recognition	Memory persistence	[70]
Mouse hippocampal HATs	HAT inhibitors	Novel object recognition	Forgetting recognition memories	[69]
Mouse NF-κB	Inhibition (κB-decoy)	Novel object recognition task	Impairing memory persistence	[69]
Rat BDNF	Blocking expression	One-trial inhibitory avoidance	Impairing memory persistence	[71]
Rat hippocampal IEGs (*c-Fos*)	Blocking de novo expression (antisense oligonucleotide)	One-trial inhibitory avoidance	Hindering persistence of LTM	[72]
Mouse hippocampal IEGs (*Arc*)	Blocking de novo expression (antisense oligonucleotide)	Context fear conditioning	Disrupted memory persistence	[73]
Rat hippocampal IEGs (*Egr-1*)	Knockdown	Inhibitory avoidance	Impairing LTM	[74]
Rat transcription factor CCAAT enhancer binding protein beta (C/EBPbeta)	Blocking expression	Inhibitory avoidance	Profound amnesia of the task	[75]
Rat nicotinic or muscarinic acetylcholine receptors (nAChR or mAChR)	Pharmacological inactivation (antagonist mecamylamine, methyllycaconitine, or scopolamine)	The step-down inhibitory avoidance learning task	Impairing fear LTM	[76,77]
Rat hippocampal beta-noradrenergic receptor	Pharmacological inactivation (blocker timolol)	Object recognition task	Impairing persistence of LTM	[78]
Rat arcaine	Administration	Contextual fear conditioning task	Impairing fear LTM	[79]
Mouse hippocampal insulin-like growth factor 2 (IGF2)	Pharmacological inactivation (anti-IGF2R antibody)	One-trial step-through inhibitory avoidance task	Attenuating the effect of IGF2 on the persistence of remote fear memories	[80]
3,4-methylenedioxyamfetamine (MDMA, ecstasy) or d-methamphetamine (METH)	Drug abuse; Administration	Neural systemic examination; Autoshaping program	Memory loss; Impairing LTM	[81,82]
Rat serotonin (5-hydroxytryptamine; 5-HT) 1A and 5-HT7 receptors	Pharmacological inhibition	Autoshaping program	Decrements in memory performance	[83]

**Table 2:** Direct biological evidence for intrinsic active forgetting.

Molecular Target	Manipulation	Experimental Paradigm	Behavioral Effect	References
*Drosophila* Rac1 (Orthologous to mammalian Rac1 and *C. elegans* ced-10)	Conditional inhibition	Pavlovian olfactory aversive conditioning	Slower early memory decay	[16]
*Drosophila Cdc42* (Orthologous to mammalian and *C. elegans* Cdc42)	Knockdown	Pavlovian olfactory aversive conditioning	Prolonged ARM	[16,86]
*Drosophila* MBdopaminergic neurons	Blocking the synaptictransmission	Pavlovian olfactory aversive/appetitive conditioning	Enhanced early memory	[17]
*Drosophila DAMB* (Limited homology with mammalian dopamine receptors)	*damb* mutation	Pavlovian olfactory aversive conditioning	Enhanced memory retention	[17]
*Drosophila Cofilin* (Orthologous to mammalian Cfl1 and *C. elegans* unc-60)	Genetic perturbation (cofilin hyperactivation)	Pavlovian olfactory aversive conditioning	Enhanced early memory performance	[16]
*Drosophila Scribble* (Orthologous to mammalian Scrib and *C. elegans* let-413)	Knockdown	Pavlovian olfactory aversive conditioning	Slower ARM decay	[19]
*Drosophila Gαq* (Gα subunits, orthologous to mammalian Gna11 and *C. elegans* egl-30)	RNAi knockdown	Pavlovian olfactory aversive conditioning	Inhibiting forgetting	[97]
*Drosophila* SCAR/WAVE Complex (Orthologous to mammalian Wasf1/Wasf13 and *C. elegans* wve-1)	RNAi	Olfactory aversive conditioning	Hampering the forgetting of early memory	[98]
*Drosophila Diaphanous* (encoding a formin family protein, orthologous to mammalian Diaph2)	RNAi	Olfactory aversive conditioning	Slower early memory decay	[98]
*Drosophila WASp* (Orthologous to mammalian Was)	Knockdown	Olfactory aversive conditioning	Increased ARM	[98]
*Drosophila* Arp2/3 Complex (Orthologous to mammalian Arpc2)	Pharmacological and genetic inhibition	Pavlovian olfactory aversive conditioning	Increased ARM	[98]
Mouse hippocampal Rac1	Activity inhibition	Novel object recognition task	Extended object recognition memory	[20]
Mouse Rac1 in nucleus accumbens (NAc)	Activity inhibition	Conditioned place preference	Patenting cocaine-induced memory performance	[23]
Mouse *Coiled-coil and C2 domain containing 1A* (*CC2D1A*, orthologous to *Drosophila* l(2)gd1)	Conditional deletion	Object location memory	Impairing object location memory	[99]
Mouse hippocampal neurogenesis	Inhibition by retroviral microinjections	Contextual fear conditioning	Mitigating forgetting	[100]
Mouse *Cdc42*	Genetic deletion	Contextual fear conditioning	Impairing remote memory recall	[101]
Rat hippocampal Rac1	Pharmacological inactivation	Contextual fear conditioning	Heightened contextual fear memory	[21]
Rat hippocampal dopamine 1-type receptors	Pharmacological inhibition	Place conditioning	Extended short-lived memory persistence	[102]
Rat hippocampal dopamine 5-type receptors	Pharmacological inactivation	Place conditioning	Extended short-lived memory persistence	[102]
*C. elegans MSI-1* (Orthologous to mammalian Msi2 and *Drosophila* Rbp6)	Genetic mutation	Olfactory conditioning	Inhibiting memory loss	[103]
*C. elegans* TIR-1/JNK-1 pathway (TIR-1 is orthologous to mammalian Sarm1 and *Drosophila* Sarm)	Genetic mutation	Olfactory adaptation and salt chemotaxis learning	Prolonged memory retention	[104]

## Data Availability

Not applicable.
